# Establishing Simultaneous T Cell Receptor Excision Circles (TREC) and K-Deleting Recombination Excision Circles (KREC) Quantification Assays and Laboratory Reference Intervals in Healthy Individuals of Different Age Groups in Hong Kong

**DOI:** 10.3389/fimmu.2020.01411

**Published:** 2020-07-16

**Authors:** Janette S. Y. Kwok, Stephen K. F. Cheung, Jenny C. Y. Ho, Ivan W. H. Tang, Patrick W. K. Chu, Eric Y. S. Leung, Pamela P. W. Lee, Daniel K. L. Cheuk, Vincent Lee, Patrick Ip, Y. L. Lau

**Affiliations:** ^1^Division of Transplantation and Immunogenetics, Department of Pathology, Queen Mary Hospital, Hong Kong, Hong Kong; ^2^Department of Paediatrics and Adolescent Medicine, The University of Hong Kong, Hong Kong, Hong Kong; ^3^Department of Paediatrics, The Chinese University of Hong Kong, Hong Kong, Hong Kong

**Keywords:** T cell receptor excision circles, K-deleting recombination excision circles, primary immunodeficiency, immune reconstitution, reference interval

## Abstract

The clinical experience gathered throughout the years has raised awareness of primary immunodeficiency diseases (PIDD). T cell receptor excision circles (TREC) and kappa-deleting recombination excision circles (KREC) assays for thymic and bone marrow outputs measurement have been widely implemented in newborn screening (NBS) programs for Severe Combined Immunodeficiency. The potential applications of combined TREC and KREC assay in PIDD diagnosis and immune reconstitution monitoring in non-neonatal patients have been suggested. Given that ethnicity, gender, and age can contribute to variations in immunity, defining the reference intervals of TREC and KREC levels in the local population is crucial for setting up cut-offs for PIDD diagnosis. In this retrospective study, 479 healthy Chinese sibling donors (240 males and 239 females; age range: 1 month−74 years) from Hong Kong were tested for TREC and KREC levels using a simultaneous quantitative real-time PCR assay. Age-specific 5^th^–95^th^ percentile reference intervals of TREC and KREC levels (expressed in copies per μL blood and copies per 10^6^ cells) were established in both pediatric and adult age groups. Significant inverse correlations between age and both TREC and KREC levels were observed in the pediatric age group. A significant higher KREC level was observed in females than males after 9–12 years of age but not for TREC. Low TREC or KREC levels were detected in patients diagnosed with mild or severe PIDD. This assay with the established local reference intervals would allow accurate diagnosis of PIDD, and potentially monitoring immune reconstitution following haematopoietic stem cell transplantation or highly active anti-retroviral therapy in the future.

## Introduction

T and B cells undergo V(D)J recombination to generate diverse and functional TCR and BCR repertoires, and these are crucial processes in the maturation of T and B cells that allow the recognition of unlimited numbers of antigens (1). During the TCR rearrangement process, excised DNA fragments create T cell receptor excision circles (TREC) that are exported to the T cell cytoplasm (2, 3). In particular, the δRec-ψJα signal joint TREC (sjTREC) is produced during TCRD deletion and detected in ~70% of (alpha-beta) αβ T cells. They are considered the most optimal target to measure the evaluation of thymic output (4–6). Kappa-deleting recombination excision circles (KREC) are produced during BCR rearrangement in naïve B cells and are analogous to TREC (7). Similarly, sjKREC formed during intronRSS-Kde rearrangements in IGK locus is a robust target for the evaluation of B cell neogenesis from bone marrow (8, 9).

Both TREC and KREC are stable and non-replicative, and are subsequently diluted during cell proliferation (10, 11). Hence, TREC and KREC analyses have been widely applied in different clinical settings to evaluate thymic and bone marrow output. Measurement of TREC and KREC levels in peripheral blood can be used for screening of Primary immunodeficiency diseases (PIDD). PIDD, also known as inborn errors of immunity (IEI), are a group of disorders that lead to defects in the development or function of the immune system. The international Union of Immunological Societies (IUIS) has classified and described over 400 PIDD (12). There is an increasing number of PIDD due to an updated definition and advancements in diagnostic technology. The disease prevalence is reported to be as high as 127 in 100,000 (13, 14), and higher rates are expected in regions where consanguinity is more common. In Hong Kong, as there is no formal registry for PIDD, the Asian Primary Immunodeficiency Network (APIN) was formed to collect data on PIDD with a mission to improve care, education, and research (15, 16). Up to January 2020, over 140 local and 750 overseas PIDD patients referred via APIN have been diagnosed at the Department of Pediatrics and Adolescent Medicine, Queen Mary Hospital, the University of Hong Kong. Pediatric patients with PIDD are more likely to have recurrent bacterial or fungal infections (17). Severe forms of PIDD such as Severe Combined Immunodeficiency (SCID) are highly fatal if diagnosis and treatment are delayed, particularly after the onset of such infections.

Since 2015, newborn screening (NBS) using TREC levels for the early identification of SCID has been implemented in Israel, New Zealand, Norway, Taiwan, Switzerland, Germany, Iceland, Italy and several regions in Canada, United States, and Australia (18–20). Recently, Sweden, Spain, and Saudi Arabia have evaluated the application of both TREC and KREC levels for screening of SCID and agammaglobulinemia (6, 21, 22). The reference intervals and cut-off values TREC and KREC have been established for neonates. Studies have demonstrated the impact of aging on thymopoiesis and bone marrow output (23, 24). In order to interpret the TREC and KREC levels for non-neonatal PIDD patients, it is necessary to establish local and ethnic reference intervals in healthy individuals of different age groups.

In this study, we measured the TREC and KREC levels of healthy Chinese individuals in Hong Kong with essential age groups of 0–18 years, as PIDD occurs in patients mainly in this age range (16, 25). We also determined the reference intervals for adults in age groups of 19–74 years for applications in thymic and/or bone marrow output monitoring for post-HSCT patients. The effects of age and gender on the KREC and TREC levels were also analyzed. The analysis of TREC and KREC levels was performed using a multiplex real-time PCR method and the values were expressed in both copies per μL blood and copies per 10^6^ cells.

## Materials and Methods

### Subjects

Archived DNA extracted from whole blood specimens from 479 healthy Chinese sibling donors aged 1 month−74 years collected during 2011–2019 for work-up for related patients requiring HSCT were used in this study. All healthy controls underwent thorough clinical evaluations and were screened for a normal blood cell count. Blood samples from 12 PIDD patients with definitive diagnosis of SCID (*n* = 2), X-SCID (*n* = 3), Agammaglobulinemia (*n* = 3), DiGeroge Syndrome (DGS, *n* = 1), and Activated PI3K-Delta Syndrome (APDS, *n* = 1), GATA2 deficiency (*n* = 1) and X-linked hyper-IgM syndrome (HIGM, *n* = 1) were used as disease controls. Reference DBS specimens were generous gifts from Dr. Francis Lee, Centers for Disease Control and Prevention (CDC), US. This study was carried out in accordance with the Declaration of Helsinki and the ICH-GCP. The protocol was approved by the Institutional Review Board of the University of Hong Kong/Hospital Authority Hong Kong West Cluster (HKU/HA HKWC IRB No. UW 18-185). The tests were performed at the Division of Transplantation and Immunogenetics, Queen Mary Hospital, Hong Kong.

### DNA Extraction From Whole Blood Specimens

Genomic DNA from EDTA whole blood samples was extracted by a magnetic beads-based purification method using the TBG EZbead blood DNA Extraction Kit (Texas BioGene Inc., Taiwan) according to the manufacturer's instructions. DNA extracted from whole blood samples (300 μL) was eluted in 100 μL Tris-EDTA buffer and stored at −70°C. DNA purity was assessed using spectrophotometer and all samples had OD260/280 ratio between 1.7 and 2.0. DNA integrity was assessed by loading 50 ng DNA in 1% agarose gel electrophoresis and no DNA degradation was detected.

### DNA Elution From Dried Blood Spot Specimens

Dried blood spot (DBS) cards were stored in low-gas permeable bags at −20°C according to Clinical and Laboratory Standards Institute (CLSI) guideline NBS06-A. DBS discs (2 mm) were punched and washed once in 100 μL Qiagen DNA elution buffer with shaking at 1,500 rpm for 10 min. The wash buffer was removed and a fresh 40 μL DNA elution buffer was added and subsequently heated at 95°C for 30 min. The eluted DNA in the supernatant was collected for TREC/KREC analysis and the TREC results were compared with those measured using CDC in-house assay. TREC/KREC analysis of 3 different spots on the same DBS sample were performed.

### Quantitative PCR Assay

Levels of TREC, KREC, and β-actin (internal control) were simultaneously quantified in a 20-μL reaction volume containing 5 μL DNA, 4 μL LightCycler Multiplex DNA Master Mix (Roche Diagnostics, Germany), 500 nM primers (TREC primer, KREC primer, and β-actin primer), and 125 nM probes (FAM-labeled TREC probe, HEX-labeled KREC probe, and Cy5-labeled β-actin probe; Integrated DNA Technologies, Singapore). The primer and probe sequences are listed in Table 1, and their designs have been previously described by Chan et al. and Sottini et al. (18, 26). The PCR analysis was performed using the LC480 II Real-Time PCR (RT-PCR) System (Roche Diagnostics, Germany) with PCR conditions of 5 min at 95°C followed by 45 cycles of 5 s at 95°C and 1 min at 60°C. The TREC/KREC plasmids were a generous gift from Dr. Sottini (26). The β-actin plasmid coding the human β-actin DNA sequence was commercially manufactured (Integrated DNA Technologies, Singapore). Standard curves for the quantification of TREC, KREC, and β-actin were obtained by using 10-fold serially diluted TREC, KREC, and β-actin plasmids (1 × 10^6^-1 × 10 copies/reaction). The copies of TREC and KREC were calculated and expressed as copies/μL blood or copies/10^6^ nucleated cells as follow:

$$\begin{array}{l} \text{TREC or KREC copies}/\mu \text{L blood}=\frac{Copy\;number\;of\;TREC\;or\;KREC\;x\;Eluted\;volume}{Blood\;volume\;for\;DNA\;extraction\;x\;5\;uL}\\ \text{ TREC or KREC copies}/10^{6}\text{ cells}=\frac{Copy\;number\;of\;TREC\;or\;KREC}{\left(Copy\;number\;of\;\beta -actin/2\right)}\;\times \;10^{6} \end{array}$$

**Table 1:** Primer and probe sequences of TREC, KREC, and β-actin.

		**Name**	**Sequence (5′->3′)**
TREC	Forward	TREC-Forward	CACATCCCTTTCAACCATGCT
	Reverse	TREC-Reverse	GCCAGCTGCAGGGTTTAGG
	Probe	TREC-FAM	ACACCTCTGGTTTTTGTAAAGGTGCCCACT
KREC	Forward	KREC-Forward	TCCCTTAGTGGCATTATTTGTATCACT
	Reverse	KREC-Reverse	AGGAGCCAGCTCTTACCCTAGAGT
	Probe	KREC-HEX	TCTGCACGGGCAGCAGGTTGG
β-actin	Forward	ACTB-Forward	ATTTCCCTCTCAGGCATGGA
	Reverse	ACTB-Reverse	CGTCACACTTCATGATGGAGTTG
	Probe	ACTB-Cy5	GTGGCATCCACGAAACTA

### Statistical Analysis

Analysis of the data was performed using the Prism program version 5.01. Data were expressed as median ± SD or range and 5^th^–95^th^ percentile for quantitative non-parametric measures, and both number and percentage for categorized data. Mann-Whitney *U*-test was used for comparisons between two independent groups for non-parametric data. Spearman's correlation coefficient test was performed to assess the strength of the relationship between studied parameters and age. A *p*-value of less than 0.05 was considered significant.

## Results

This study included 294 healthy pediatric sibling donors (150 males and 144 females; age range: 1 month−18 years; median age: 9.9 years) and 185 adult donors (90 males and 95 females; median age: 44.1 years). The number of males and females in the different age groups are listed in Table 2. The TREC and KREC levels were calculated as copies/μL blood and copies/10^6^ cells. The medians and ranges of TREC and KREC levels of male and female in different age groups were provided in the Supplementary Tables 1, 2. Samples with TREC and KREC levels below the detection limit (10 copies/reaction) were checked for the presence of β-actin copies to confirm that the low levels were not due to amplification errors and therefore amplification failure can be ruled out.

**Table 2:** Number of subjects in different age groups.

**Age (years)**	**Male (*n*)**	**Female (*n*)**	**Total**
<1	5	4	9
1–4	32	25	57
5–8	37	30	67
9–12	25	34	59
13–18	51	51	102
19–30	20	18	38
31–40	19	24	43
41–50	16	15	31
51–60	16	15	31
>61	19	24	43
Total	240 (50%)	239 (50%)	479

### TREC and KREC Levels Declined With Increasing Age

**Figure 1:**
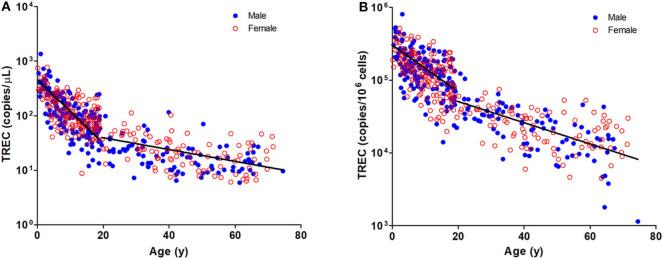
Dot Plot showing TREC copies/μL blood **(A)** and TREC copies/10^6^ cells **(B)** among the study age groups. Blue full circles represent healthy males and red empty circles represent healthy females. A significant inverse correlation was observed between TREC levels and both pediatric and adult age groups (Pediatric: r = −0.6488, *p* < 0.0001 for copies/μL and r = −0.5487, *p* < 0.0001 for copies/10^6^ cells; Adult: r = −0.4924, *p* < 0.0001 for copies/μL and r = −0.6289, *p* < 0.0001 for copies/10^6^ cells).

**Figure 2:**
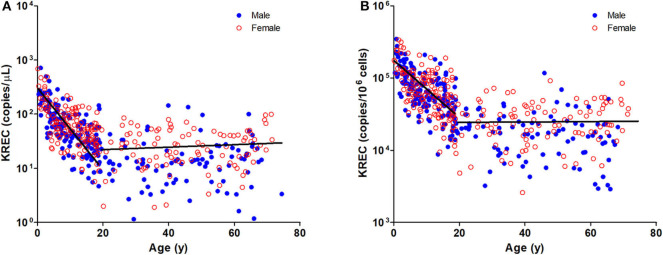
Dot Plot showing KREC copies/μL blood **(A)** and TREC copies/10^6^ cells **(B)** among the study age groups. Blue full circles represent healthy males and red empty circles represent healthy females. A significant inverse correlation was observed between KREC levels and pediatric age groups (Pediatric: r = −0.6577, *p* < 0.0001 for copies/μL and r = −0.6241, *p* < 0.0001 for copies/10^6^ cells; Adult: r = 0.0903, *p* = 0.2216 for copies/μL and r = −0.0171, *p* = 0.8176 for copies/10^6^ cells).

**Figure 3:**
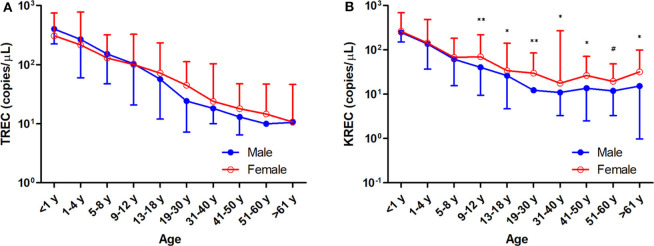
Trend of TREC copies/μL blood **(A)** and KREC copies/μL blood **(B)** among the study age groups. Data is expressed as median ± range. Blue full circles represent healthy males and red empty circles represent healthy females. A significant higher KREC level was observed for age groups after 9–12 years, except for 51–60 years. ^*^p < 0.05, ^**^p < 0.01, ^#^*p* = 0.0510.

**Figure 4:**
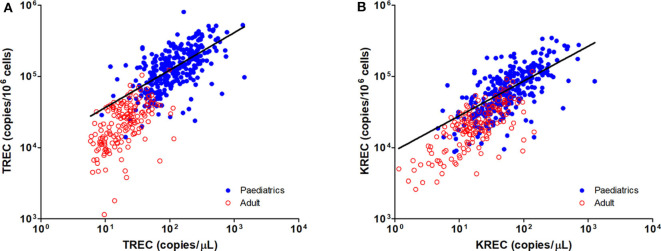
Dot Plot showing the correlations between copies/μL blood and copies/10^6^ cells for TREC **(A)** and KREC **(B)** levels. Blue full circles represent healthy pediatric individuals and red empty circles represent healthy adults. A significant positive correlation was observed between both units for TREC and KREC levels (TREC level: r = 0.8319, *p* < 0.0001; KREC level: r = 0.7794, *p* < 0.0001).

The TREC and KREC levels were plotted against age of healthy individuals to assess any overall correlations (Figures 1, 2). A distinct pattern was observed in pediatric individuals (0–18 years) compared to adults (>18 years), thus the data was divided into pediatric and adult age groups and analyzed separately. The highest levels of TREC and KREC were observed in those of early age. There was a significant inverse correlation between TREC levels (both copies/μL blood and copies/10^6^ cells) and age for both pediatric and adult groups. The rate of decline in TREC level with age was greater in the pediatric group and slowed down with age in the adult group. A significant inverse correlation between KREC level (both copies/μL blood and copies/10^6^ cells) was also found in the pediatric group, but not in the adult group. The KREC level varied but was maintained at a stably low level in adult. No difference in TREC level was observed between males and females in all age groups. A significantly higher KREC level (copies/μL blood) was detected in females than in males after 9–12 years of age (Figure 3). In addition, a significant positive correlation was observed between units in copies/μL blood and units in copies/10^6^ cells for both TREC and KREC levels (Figure 4).

### Reference Intervals of Healthy Individuals for Different Age Group

To establish reference intervals for TREC and KREC levels in the Hong Kong Chinese population, subjects were divided into 10 different age groups (5 groups for pediatric age and 5 groups for adults) and reference intervals were expressed as median and 5^th^–95^th^ percentile range (Table 3). The lower threshold (5^th^ percentile) of reference ranges for TREC (copies/μL blood and copies/10^6^ cells) in pediatric age groups were 223 and 151,107 (<1 year), 74 and 66,845 (1–4 years), 53 and 58,281 (5–8 years), 30 and 38,206 (9–12 years), 21 and 27,173 (13–18 year); and in adult groups were 12 and 20,831 (19–30 years), 10 and 8,436 (31–40 years), 7 and 6,644 (41–50 years), 0 and 0 (51–60 year), and 0 and 0 (>61 years). The lower threshold (5^th^ percentile) of reference ranges for KREC (copies/μL blood and copies/10^6^ cells) in pediatric age groups were 134 and 115,946 (<1 year), 31 and 35,491 (1–4 years), 21 and 29,471 (5–8 years), 16 and 25,425 (9–12 years), 8 and 11,684 (13–18 years); and in adult groups were 1 and 3,063 (19–30 years), 2 and 6,098 (31–40 years), 4 and 7,363 (41–50 years), 3 and 5,566 (51–60 years) and 1 and 2,907 (>61 years).

**Table 3:** Reference intervals of TREC and KREC in different age groups.

**Age group (years)**	***n***	**TREC (copies/μL)**		**TREC (copies/10**^****6****^ **cells)**		**KREC (copies/μL)**		**KREC (copies/10**^****6****^ **cells)**	
		**Median ± SD**	**5^**th**^–95^**th**^ percentile**	**Median ± SD**	**5^**th**^–95^**th**^ percentile**	**Median ± SD**	**5^**th**^–95^**th**^ percentile**	**Median ± SD**	**5^**th**^–95^**th**^ percentile**
<1	9	313 ± 363	223–1,355	304,896 ± 136,873	151,107–526,408	249 ± 219	134–713	217,210 ± 74,795	115,946–345,920
1–4	57	249 ± 170	74–656	213,155 ± 134,031	66,845–461,833	140 ± 116	31–470	114,289 ± 69,069	35,491–275,571
5–8	67	144 ± 83	53–284	151,838 ± 84,272	58,281–357,873	65 ± 47	21–171	69,186 ± 35,861	29,471–150,023
9–12	59	101 ± 73	30–279	125,197 ± 68,390	38,206–285,532	51 ± 46	16–186	64,424 ± 40,532	25,425–182,274
13–18	102	64 ± 50	21–209	86,770 ± 58,610	27,173–229,987	30 ± 28	8–107	36,610 ± 33,621	11,684–116,963
19–30	38	29 ± 22	12–99	38,543 ± 20,899	20,831–104,674	19 ± 20	1–70	21,262 ± 15,840	3,063–66,698
31–40	43	22 ± 23	10–96	29,356 ± 15,133	8,436–61,274	14 ± 28	2–77	18,056 ± 14,683	6,098–59,155
41–50	31	17 ± 13	7–57	20,996 ± 10,369	6,644–40,467	16 ± 29	4–114	22,835 ± 23,488	7,363–103,165
51–60	31	11 ± 10	0–37	12,706 ± 14,525	0–52,945	13 ± 13	3–45	17,933 ± 15,506	5,566–58,917
>61	43	11 ± 12	0–39	11,668 ± 11,130	0–35,331	22 ± 26	1–99	21,092 ± 18,671	2,907–53,655

### Validation of Assay With Reference DBS Specimens

DNA from the 21 reference DBS specimens were eluted and tested using the TREC and KREC assays (Table 4). “Expected” TREC levels of the reference samples measured using an in-house RT-PCR TREC assay were provided by Dr. Francis Lee, CDC, US. Beta-actin (internal control) was detected in all DBS specimens except the negative control that was prepared from leukocyte-depleted blood and served as a negative control. Out of the 15 DBS specimens prepared from normal cord blood (within TREC reference range), we detected positive TREC levels ranging from 144 to 514 copies/μL blood. For the five SCID-like DBS samples prepared from PBMC-depleted blood, TREC levels were all below the detection limit. These results were in concordance with the expected results, and the TREC levels measured using our assay were highly correlated with those measured by the CDC (Supplementary Figure 1). On the other hand, there was no reference KREC samples available, no comparison could be done.

**Table 4:** Comparison of TREC results with reference DBS specimens.

		**Reference results**			**Total**
		**Normal (TREC +ve)**	**SCID-like (TREC –ve)**	**Unsatisfactory sample (reference gene out of range)**	
**Measured results**	TREC +ve ACTB +ve	15	0	0	**15**
	TREC –ve ACTB +ve	0	5	0	**5**
	TREC –ve ACTB –ve	0	0	1	**1**
	**Total**	**15**	**5**	**1**	**21**

### Use of TREC and KREC Assays in PIDD Patients

Blood samples from patients diagnosed with PIDD were tested with the TREC and KREC assays and compared with their age-matched reference intervals. The results and genetic diagnosis of the patients are listed in Table 5. Patient P1 presented with recurrent infections as well as low CD4+ T cell and CD19+ B cell counts. The patient was diagnosed with compound heterozygous mutations in RAG1 and both TREC and KREC levels were very low or undetectable. Patient P2 has a history of severe chest infection, profound T cell lymphopenia and borderline low B cell count. The patient was diagnosed with compound heterozygous mutations in IL7RA and Diffuse Large B cell Lymphoma (DLBCL). The patient had much lower TREC and KREC levels than the age-matched reference intervals. Patients P3, P4, and P5 were classical T^−^B^+^NK^+^ X-linked SCID patients with IL2RG defects. They all had undetectable TREC which was in agreement with their low T cell counts. Patient P5 had lower KREC level which could be explained by the low B cell count. Patient P6 was diagnosed with partial DGS, and had low TREC and normal KREC levels compared with the corresponding age group. Patient P7 was diagnosed with APDS, and had both lower TREC and KREC levels compared with the age-matched reference interval which were in concordance with the low T and B cells counts. Patients P8 and P9 were both diagnosed with XLA and had defects in BTK gene. Patient P10 had agammaglobulinemia phenotype with unknown genetic cause as found by Whole Exome Sequencing while patient P11 was diagnosed with GATA2 deficiency. Patients P8, P9, P10, and P11 were T^+^B^−^ PIDD and they all had normal TREC but very low KREC levels. Patient P12 was diagnosed with HIGM and the TREC and KREC levels were normal which were consistent with normal T and B cell counts. The 5^th^ percentile lower thresholds of TREC and KREC were able to detect severe and mild abnormalities in the thymic function and B cell neogenesis in these patients.

**Table 5:** Results of TREC and KREC levels in patients with primary immunodeficiency diseases.

**Patient**	**Age**	**Diagnosis**	**Genetic diagnosis**	**TREC (copies/μL)**	**TREC (copies/10^**6**^ cells)**	**KREC (copies/μL)**	**KREC (copies/10^**6**^ cells)**	**Lymphocytes subset (cells/μL)**	**IgG/A/M (mg/dL)**
P1	<1 y	SCID	RAG1 (c.322C>T & c.2095C>T)	* < LOD L*	* < LOD L*	*1 L*	*2,101 L*	CD3: 1,474, CD4: 531,CD8: 962; CD19: 226	IgG: 1,990,IgA: 33, IgM: 374
P2	<1 y	SCID; DLBCL	IL7RA (c.221+2T>A & c.361dupA)	* < LOD L*	* < LOD L*	*2 L*	*3,946 L*	CD3: 81, CD4: 7,CD8: 66; CD19: 700	IgG: 1,008,IgA:123, IgM: 181
P3	<1 y	X-SCID	IL2RG (c.694G>C)	* < LOD L*	* < LOD L*	*136*	*187,993*	CD3: 292, CD4: 31,CD8: 141, CD19: 910	IgG: 755,IgA: <7, IgM: 11
P4	<1 y	X-SCID	IL2RG (c.996C>T)	* < LOD L*	* < LOD L*	*142*	*61,424 L*	Not available	Not available
P5	<1 y	X-SCID	IL2RG (c.723T>G)	* < LOD L*	* < LOD L*	*43 L*	*26,158 L*	CD3: 175, CD4: 33,CD8: 136; CD19: 240	IgG: 326,IgA: <7, IgM: <5
P6	<1 y	DiGeorge Syndrome	22q11.21 deletion	*58 L*	*33,296 L*	138	*79,781 L*	CD3: 1,850, CD4: 1,200,CD8: 537; CD19: 892	IgG: 761,IgA: <10, IgM: <20
P7	5–8 y	APDS	PIK3CD (c.3061G>A)	*37 L*	*51,175 L*	*14 L*	*19,414 L*	CD3: 1,594, CD4: 831,CD8: 706; CD19: 98	IgG: 2,658,IgA: 77, IgM: 914
P8	1–4 y	XLA	BTK (c.1723G>C)	141	*61,379 L*	* < LOD L*	* < LOD L*	CD3: 2,212, CD4: 1,241,CD8: 674; CD19: 15	IgG: 336,IgA: <7, IgM: 18
P9	13–18 y	XLA	BTK (c.1535T>C)	87	76,075	*1 L*	*791 L*	CD3: 2,399, CD4: 927,CD8: 1,381; CD19: 8	IgG: <75,IgA: <10, IgM: 20
P10	<1 y	Agamma-globulinemia	Unknown	295	*88,500 L*	* < LOD L*	* < LOD L*	CD3: 7,845, CD4: 3,437,CD8: 5,116; CD19: 13	IgG: 726,IgA: <7, IgM: 27
P11	13–18 y	GATA2 deficiency; Monosomy 7	GATA2 (c.726_729del)	52	74,876	* < LOD L*	* < LOD L*	CD3: 1,169, CD4: 525,CD8: 599; CD19: 33	IgG: 766,IgA: 58, IgM: 106
P12	5–8 y	HIGM	CD40LG (c.761G>T)	175	83,734	109	51,992	CD3: 3,560, CD4: 1,995,CD8: 957; CD19: 767	IgG: 298,IgA: 61, IgM: 185

*SCID, Severe Combined Immunodeficiency; DLBCL, Diffuse large B-cell lymphoma; X-SCID, X-linked SCID; XLA, X-linked Agammaglobulinemia; APDS, Activated PI3K-Delta Syndrome (APDS); HIGM, X-linked immunodeficiency with hyper-IgM syndrome; LOD, Limit of Detection; L, Below reference interval*.

## Discussion

To the best of our knowledge, this is the first and largest study to determine TREC and KREC reference levels in healthy Chinese pediatric and adult individuals. A total of 479 healthy individuals were divided into 10 different age groups for calculating the age-specific reference intervals, with the lower threshold defined as the 5^th^ percentile. The pediatrics age ranges were selected with reference to previous studies (26, 27). We also determined the reference intervals for adults in age groups between 19 and 74 years for the potential applications in thymic and/or bone marrow output monitoring for post-HSCT patients and HIV patients.

We and other groups have observed a significant inverse correlation of TREC levels with increasing age (28–31). A greater downward trend was observed in the pediatric group than in adults, which is in agreement with other studies (28, 32). We detected the highest TREC and KREC levels in infants <1 year old (33, 34), which may be explained by the continual thymic development in the first year of life (35, 36). We found the TREC levels decreased with age, which is suggested to be related to thymic involution (37), and lower TREC and KREC levels result from dilution during homeostatic replication of T or B memory cells or antigen-induced proliferation (9, 38). In contrast to the steady decline of TREC levels in the adult age group, KREC levels remained at a relatively stable level indicating sustainable B cell neogenesis throughout life, which was in agreement with the findings reported previously (26, 39). The rapid decline of TREC and KREC levels in pediatrics emphasized the importance of establishing the age-specific reference intervals for PIDD diagnosis as the patients occurred mainly in this age range (16, 25).

Our findings showed no significant differences in TREC levels between females and males, regardless of age, which echoed some studies that also found no differences between genders (32, 40, 41). However, several studies observed a higher TREC level in female adolescents and a slower decline of TREC levels in adult females compared to males (26, 28, 42), and Rechavi et al. reported a higher TREC level in female neonates than in male neonates (43). Interestingly, we observed a significantly higher level of KREC in females than males after 9–12 years (except for the 51–60 year age group), indicating a slower decline in KREC levels. This may be explained by the substantial immunomodulatory role of sex hormones on immune responses (44, 45). However, differences in the genetic backgrounds of ethnic groups may contribute to the contradictory findings. A larger sample size of infant healthy controls (<1 year) is needed to elucidate differences in TREC and KREC levels taking into account these factors. Moreover, TREC levels are maintained at very low levels or even undetectable in older age due to physiological decline of thymic function in the fifth and sixth decades of life, which might suggest that this assay is not suitable for adults over 50 years.

The diagnosis of PIDD is usually delayed until presentation of clinical symptoms, such as recurrent infections. The conventional tests for diagnosis of PIDD include total lymphocyte count, lymphocyte subset, immunoglobulin measurement, functional assays, and genetic analysis. The latter two remain the most important tests and are critical for diagnosis. TREC and KREC analysis is a fast, cost-effective and sensitive tool to for SCID screening and PIDD diagnosis, especially as only a small sample volume is needed for DNA extraction which minimizes harm to young age infants. It may not be feasible to perform all the conventional tests if sufficient blood is not available. The protocols of TREC and KREC assay varies in laboratories, and the genetic difference in populations may also contribute to the variation of cut-off in NBS programs in different countries. Therefore, the age- and ethnicity-matched TREC and KREC reference intervals reported in this study are crucial for the diagnosis of PIDD in young children and adolescents in the local population. Nevertheless, PIDD is a rare disease with around 140 cases diagnosed in Hong Kong in the past 30 years (46–48). Implementation of TREC (and KREC) based NBS program with local cut-off can greatly facilitate the early identification of severe PIDD patients. A quicker diagnosis allows earlier genotype-specific treatments (e.g., HSCT) prior to life-threatening infections, which offers a better outcome and improves the quality of life (49–51). This will also significantly reduce the costs of delayed diagnosis and treatment of related morbidities (52–54). We have shown a good correlation between the TREC results using our established assay with the CDC in-house method, indicating this assay is potentially applicable for the NBS program for severe PIDD.

Our TREC and KREC assay was able to correctly identify the 11 selected PIDD patients with aberrant T and/or B cell counts. The findings for SCID (RAG1 and IL2RG), XLA and GATA2 deficiency are in agreement with previous reports (55–59). Typical defect in IL7RA results in a phenotype of T^−^B^+^NK^+^. However, the patient P2 in this study has concomitant EBV associated DLBCL. This might explain the low KREC level even the patient had a CD19 count of 600/μL and the circulating B cells might be dominated by the monoclonal expansion of cancer cells. Borte et al. also reported that patients with IL7RA defects could have variable KREC levels with the lowest level at ~10 copies/μL (55). Recent pilot KREC NBS studies and our study have demonstrated the feasibility in detecting XLA and agammaglobulinemia (6, 60, 61). The identification of DGS has been challenging as the phenotypes vary from “partial” to “complete” reduction of thymus. Fronkova et al. reported that DGS patients had significantly lower TREC levels compared with controls, whereas TREC and KREC levels in most patients were within normal ranges (62). In our study, we detected a low TREC level and normal KREC level in a partial DGS patient compared with their age-matched 5^th^ percentile reference interval. Gul et al. and Liao et al. also found their DGS patients had TREC levels below the 5^th^ percentile and <90 copies, respectively (63, 64). Moreover, our assay was able to detect APDS caused by gain of function in PIK3CD which is a common variable immunodeficiency (CVID) with low TREC and KREC levels (65). As expected, HIGM patient with CD40LG defect has TREC and KREC levels within normal ranges (55). The application of 5^th^ percentile for TREC and KREC levels as the lower cut-off allows the detection of milder forms of T and B cells lymphopenia in PIDD diagnosis. Therefore, TREC and KREC assay is a valuable tool to facilitate the diagnosis of PIDD, in combination with other laboratory tests, such as lymphocyte subset and immunoglobulin measurement.

Since the first use of TREC level in determining thymic output by Douek et al., the units of measurement for TREC level have varied, including TREC/μg DNA of T cells, TREC/CD45RA+ T cell, TREC/10^5^ CD4+ T cells, TREC/10^6^ PBMCs, and TREC/mL or μL of blood (19, 28, 66). In this study, we measured the TREC, KREC, and β-actin (internal control) copy number using a multiplex RT-PCR assay and presented the TREC and KREC levels using two methods. We first aligned the formula used for the NBS program and expressed the levels in copies/μL blood (18, 19, 67). The TREC and KREC levels were then normalized with β-actin to calculate the levels in copies/10^6^ cells (26). We showed a high correlation between the two units for both TREC and KREC levels. These calculation approaches avoided the need for flow cytometry to estimate the numbers of T cells or differential WBC counts, as the values may not be always available. The TREC and KREC levels in copies/μL of blood is suggested to be a better estimation of new lymphocyte maturation regardless of homeostatic cell replication (67). However, patients with lymphopenia due to immunosuppressive treatments may show “false positives” of low TREC and KREC copies per blood volume (6). In addition, patients with high T cells lymphoproliferation or monoclonal B cells proliferation may produce low TREC or KREC levels due to dilution in maturing cells (68). The TREC and KREC levels in copies/10^6^ cells were normalized with the cell number in blood, which provides a more accurate evaluation of T and B cell function in such cases. However, elevated neutrophil numbers (e.g., during infection) may lead to under-estimation of TREC and KREC levels when using copies/10^6^ cells. Therefore, TREC and KREC levels measured in both units should be interpreted with care and other clinical information should be reviewed. Quantification of TREC levels can reflect the thymic output, as it is present in new and recent maturing lymphocytes, but low levels of TREC can still remain in some long-lived naïve cells, which can lead to over-estimation in the results (5, 69, 70).

Multiple methods have been applied for the quantitative measurement of TREC and/or KREC including RT-PCR, which is one of the most commonly used and cost-effective technologies (55). The introduction of KREC measurement offers an additional tool for detecting B cell lymphopenia. On the other hand, multiplex TREC, KREC and internal control in a single reaction using RT-PCR eliminates the variability associated with pipetting errors and allows accurate evaluation of TREC and KREC levels in a cost-effective manner (71). Pilot NBS study using TREC and KREC has also demonstrated improved diagnostic rates for severe PIDD (6, 60). Recent developments have also included the detection of exon 7 of SMN1 in a single reaction for the diagnostic testing of Spinal Muscular Atrophy (72–74). The use of digital PCR technology offers even better limits of detection and higher precision, which is particularly useful for samples with low copy numbers to help reduce the false positive rate (75–77). However, the drawback is the higher instrument costs (91, 93). Measurement of donor cell engraftment using chimerism analysis is a standard tool to determine immunity reconstitution (61). The TREC and KREC assay has been applied in post-HSCT monitoring of functional reconstitution of T and B cells (78, 79). The results obtained in this study could serve as age-matched reference intervals for the comparison of adult patients. Further study with more clinical samples for such applications is ongoing.

The findings in this study need to be interpreted with the following caveats. First, the number of archived samples for healthy infants (<1 year) was limited, which constitutes the major drawback of this study. This may be overcome by recruitment of more healthy subjects. This study also lacked adult PIDD samples for the evaluation of reference intervals in certain adult age groups. An international quality assurance program is available only for TREC measurements, thus an equivalent program for KREC measurements is warranted for standardizing assay performance across different laboratories (80).

## Conclusion

To the best of our knowledge, we are first to report reference intervals of TREC and KREC levels from the largest sample size of healthy individuals in a Chinese population. Our study demonstrated that TREC and KREC levels decline with age, which is an important factor for the accurate measurement of TREC and KREC levels. This study generated age-matched reference values that allow us to interpret and compare results in samples of children, adolescents, and adults with suspected compromised immunity. The quantification of TREC and KREC levels simultaneously obtained by RT-PCR can be easily introduced into routine laboratory practice and is highly informative. PIDD including SCID, X-SCID, XLA, partial DGS, GATA2 deficiency, and APDS were successfully diagnosed using this assay. This assay is fast and cost-effective and the established age-specific reference intervals can be applied as a diagnostic tool for PIDD.

## Data Availability Statement

All datasets presented in this study are included in the article/Supplementary Material.

## Ethics Statement

The studies involving human participants were reviewed and approved by Institutional Review Board of the University of Hong Kong/Hospital Authority Hong Kong West Cluster (HKU/HA HKW IRB). Written, informed consent was obtained from the individuals and/or minors' legal guardian/next of kin for the publication of any potentially identifiable images or data included in this article.

## Author Contributions

The study was designed by JK, JH, SC, and PL. The TREC/KREC assays were developed by JH, SC, and IT. The samples were provided by PL, YL, DC, and VL. The TREC/KREC assays were performed by IT, PC, and EL. Data were collated by JH, SC, EL, and JK and analyzed by SC and JK. Statistical analyses were carried out by SC, EL, and JK. The manuscript was written by SC, PI, YL, and JK. The final manuscript was reviewed by all authors.

## Conflict of Interest

The authors declare that the research was conducted in the absence of any commercial or financial relationships that could be construed as a potential conflict of interest. The handling editor declared a past co-authorship with one of the authors YL.

## Supplementary Material

Supplementary Figure 1 Dot Plot comparing the TREC copies/μL blood from measured results and expected results provided by the CDC for reference DBS specimens. A significant and strong positive correlation was observed between both methods (r = 0. 9176, *p* < 0.0001).
