# Supplementary figures and images for: Establishing Simultaneous T Cell Receptor Excision Circles (TREC) and K-Deleting Recombination Excision Circles (KREC) Quantification Assays and Laboratory Reference Intervals in Healthy Individuals of Different Age Groups in Hong Kong
Source: Front Immunol. 2020 Jul 16;11:1411. doi: 10.3389/fimmu.2020.01411 (PMC7378446; doi:10.3389/fimmu.2020.01411)

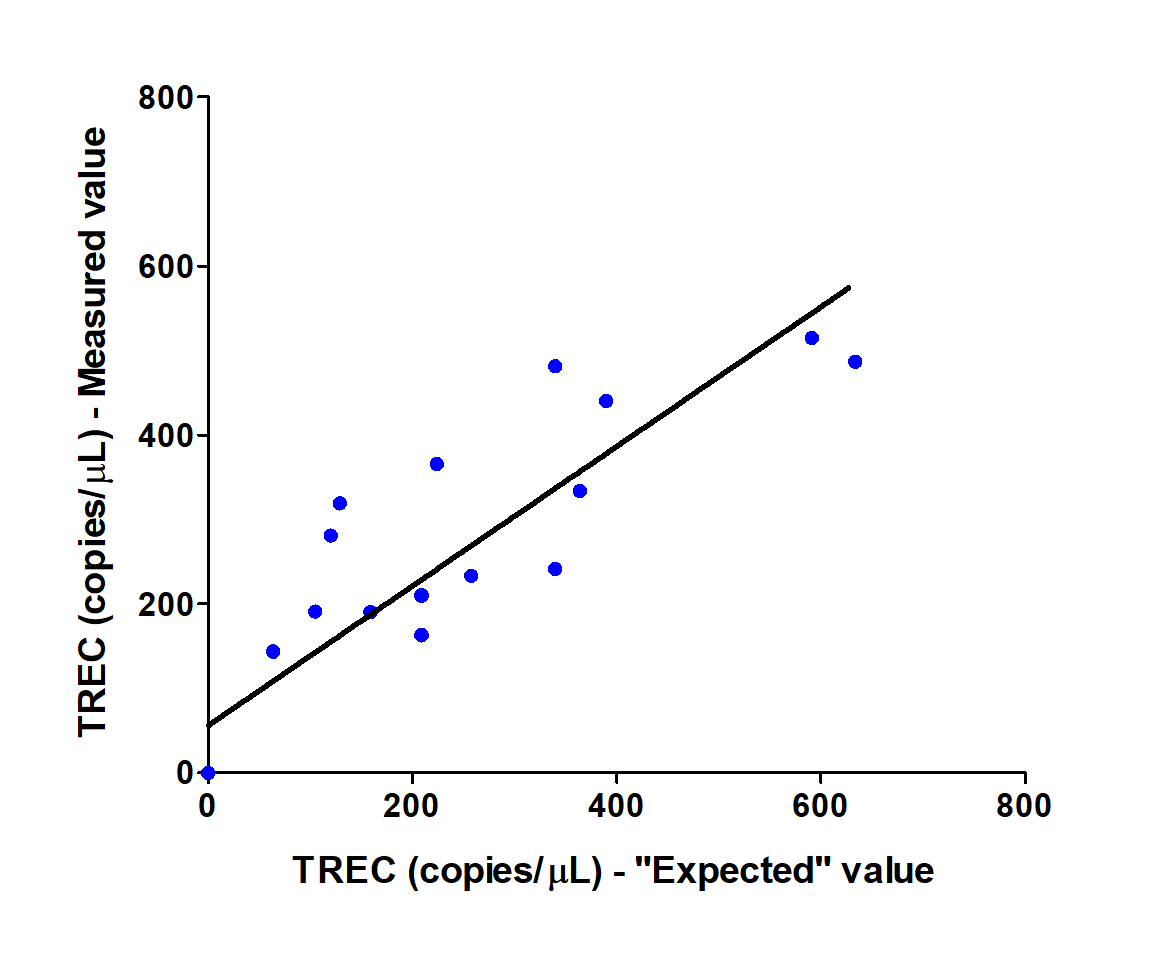

Supplement: Supplementary Figure 1 — Dot Plot comparing the TREC copies/μL blood from measured results and expected results provided by the CDC for reference DBS specimens. A significant and strong positive correlation was observed between both methods (r = 0. 9176, p < 0.0001). [file Image_1.tif]
