# Supplementary material for: Establishing Simultaneous T Cell Receptor Excision Circles (TREC) and K-Deleting Recombination Excision Circles (KREC) Quantification Assays and Laboratory Reference Intervals in Healthy Individuals of Different Age Groups in Hong Kong
Source: Front Immunol. 2020 Jul 16;11:1411. doi: 10.3389/fimmu.2020.01411 (PMC7378446; doi:10.3389/fimmu.2020.01411)
Supplement: Supplementary file 2 [file Data_Sheet_1.docx]

**Supplementary Table 1 Median** ± **SD of TREC and KREC in different age groups and genders**

| Age group | n | | TREC (copies/µL) | | TREC (copies/10^6^ cells) | | KREC (copies/µL) | | KREC (copies/10^6^ cells) | |
| --- | --- | --- | --- | --- | --- | --- | --- | --- | --- | --- |
|  | Male | Female | Male | Female | Male | Female | Male | Female | Male | Female |
| <1 y | 5 | 4 | 400 ± 463 | 308 ± 221 | 389,273 ± 135,977 | 225,934 ± 104,191 | 249 ± 222 | 263 ± 248 | 230,186 ± 83,711 | 172,033 ± 52,512 |
| 1-4 y | 32 | 25 | 267 ± 169 | 216 ± 173 | 203,614 ± 151,482 | 213,996 ± 110,594 | 136 ± 102 | 143 ± 134 | 107,165 ± 49,662 | 119,820 ± 86,570 |
| 5-8 y | 37 | 30 | 152 ± 84 | 130 ± 83 | 151,298 ± 76,022 | 157,892 ± 94,067 | 62 ± 53 | 67 ± 37 | 65,811 ± 33,159 | 73,049 ± 39,189 |
| 9-12 y | 25 | 34 | 103 ± 69 | 101 ± 75 | 142,829 ± 69,336 | 118,050 ± 67,770 | 40 ± 39 | 70 ± 48 | 55,184 ± 46,253 | 74,104 ± 36,124 |
| 13-18 y | 51 | 51 | 57 ± 49 | 72 ± 50 | 86,856 ± 58,184 | 86,685 ± 59,612 | 26 ± 29 | 34 ± 28 | 40,031 ± 32,051 | 33,269 ± 35,417 |
| 19-30 y | 20 | 18 | 24 ± 19 | 45 ± 23 | 34,025 ± 26,460 | 39,464 ± 12,381 | 13 ± 11 | 30 ± 23 | 20,435 ± 10,159 | 27,631 ± 18,818 |
| 31-40 y | 19 | 24 | 18 ± 26 | 24 ± 20 | 32,791 ± 15,516 | 26,985 ± 14,878 | 11 ± 16 | 17 ± 22 | 16,741 ± 11,301 | 20,843 ± 16,648 |
| 41-50 y | 16 | 15 | 13 ± 15 | 18 ± 10 | 21,588 ± 9,745 | 18,112 ± 11,387 | 14 ± 24 | 27 ± 17 | 17,990 ± 26,750 | 26,393 ± 19,871 |
| 51-60y | 16 | 15 | 10 ± 9 | 15 ± 11 | 9,467 ± 12,179 | 13,376 ± 16,270 | 12 ± 9 | 22 ± 15 | 17,740 ± 17,095 | 17,933 ± 14,215 |
| >61 yr | 19 | 24 | 11 ± 9 | 11 ± 14 | 7,757 ± 10,277 | 14,214 ± 11,324 | 15 ± 23 | 32 ± 26 | 12,799 ± 11,766 | 34,989 ± 18,072 |

**Supplementary Table 2 Range of TREC and KREC in different age groups and genders**

| Age group | n | | TREC (copies/µL) | | TREC (copies/10^6^ cells) | | KREC (copies/µL) | | KREC (copies/10^6^ cells) | |
| --- | --- | --- | --- | --- | --- | --- | --- | --- | --- | --- |
|  | Male | Female | Male | Female | Male | Female | Male | Female | Male | Female |
| <1 y | 5 | 4 | 223 - 1355 | 295 - 746 | 206,425 - 526,408 | 151,106 - 388,100 | 152 - 713 | 134 - 689 | 118,080 - 345,920 | 115,946 - 243,897 |
| 1-4 y | 32 | 25 | 60 - 726 | 41 - 773 | 56,788 - 803,727 | 59,226 - 513,173 | 37 - 496 | 23 - 485 | 30,449 - 219,649 | 33,160 - 336,015 |
| 5-8 y | 37 | 30 | 48 - 440 | 51 - 319 | 52,465 - 346,213 | 45,028 - 400,607 | 16 - 287 | 19 - 181 | 24,640 - 160,591 | 25,993 - 207,759 |
| 9-12 y | 25 | 34 | 21 - 279 | 39 - 327 | 33,583 - 285,532 | 29,193 - 336,968 | 9 - 186 | 17 - 219 | 17,629 - 209,234 | 20,335 - 191,911 |
| 13-18 y | 51 | 51 | 12 - 247 | 9 - 234 | 14,047 - 247,158 | 19,752 - 239,767 | 5 - 145 | 9 - 141 | 8,737 - 158,235 | 9,029 - 189,099 |
| 19-30 y | 20 | 18 | 7 - 98 | 13 - 113 | 21,214 - 122,338 | 13,559 - 66,801 | 1 - 42 | 2 - 85 | 3,225 - 38,506 | 4,813 - 72,244 |
| 31-40 y | 19 | 24 | 10 - 116 | 8 - 103 | 8,214 - 64,201 | 5,992 - 74,816 | 3 - 58 | 2 - 80 | 5,984 - 44,277 | 2,587 - 65,068 |
| 41-50 y | 16 | 15 | 6 - 70 | 8 - 48 | 6,490 - 39,049 | 8,166 - 42,200 | 3 - 97 | 13 - 71 | 4,297 - 117,353 | 11,137 - 91,557 |
| 51-60y | 16 | 15 | 0 - 28 | 0 - 47 | 0 - 45,766 | 0 - 53,095 | 3 - 28 | 7 - 48 | 5,453 - 69,231 | 5,976 - 52,041 |
| >61 yr | 19 | 24 | 0 - 28 | 0 - 46 | 0 - 42,828 | 0 - 35,989 | 1 - 100 | 4 - 100 | 395 - 51,116 | 6,095 - 84,574 |
